# Recurrent spinal IgG4-related hypertrophic pachymeningitis and its management: a case report

**DOI:** 10.3389/fimmu.2026.1778359

**Published:** 2026-05-05

**Authors:** Gorbachev Jowah, Tzuen Kao, Yew-Weng Fong

**Affiliations:** 1 University of Iowa Hospitals and Clinics, Iowa City, IA, United States; 2 Division of Neurosurgery, Department of Surgery, Cathay General Hospital, Taipei, Taiwan; 3 Department of Medicine, School of Medicine, Fu Jen Catholic University, New Taipei City, Taiwan

**Keywords:** azathioprine, IgG4-related disease, recurrence, spinal epidural tumor, steroid therapy, treatment response

## Abstract

**Aim:**

In this report, we share a case of recurrent spinal immunoglobulin G4 (IgG4)-related hypertrophic pachymeningitis (IgG4-RHP) in a patient who initially achieved near-complete recovery of muscle weakness after decompression surgery and steroid therapy. However, the patient experienced disease recurrence after discontinuing steroid therapy due to intolerable side effects.

**Materials and methods:**

We reviewed the patient’s medical records, including the pathology images and report certified by the pathologist. After disease recurrence, the patient was treated with oral prednisolone 5 mg twice daily and oral azathioprine 50 mg daily initially, which was then increased to 75 mg daily a month later.

**Results:**

After 6 months of combined therapy with prednisolone and azathioprine, there was significant improvement in the patient’s clinical symptoms. Interval MRI scans showed resolution of the lesion, and the serum IgG4 concentration also returned to the normal range.

**Conclusion:**

Although the pachymeninges (dura mater) are rarely involved in IgG4-related disease, it should be considered in the differential diagnosis of long-segment, homogeneously enhanced epidural masses. Steroid therapy is the first-line therapy for IgG4-RHP, while steroid-sparing agents should be added in refractory or recurrent cases. Surgical decompression is necessary in cases of acute neurological deterioration. A combination of a steroid and an immunomodulatory agent may be considered in cases of recurrent disease. More importantly, we demonstrated details of the clinical course of IgG4-RHP and the treatment effects of both surgical decompression of the spinal cord and medical treatment with steroids in combination with an immunomodulator (azathioprine), particularly in the setting of disease recurrence. This was done in full detail with interval imaging scans showing the response to treatment, along with serial tracking of the antibody levels, which, to our knowledge, had not been reported prior.

## Case report

**Figure 1:**
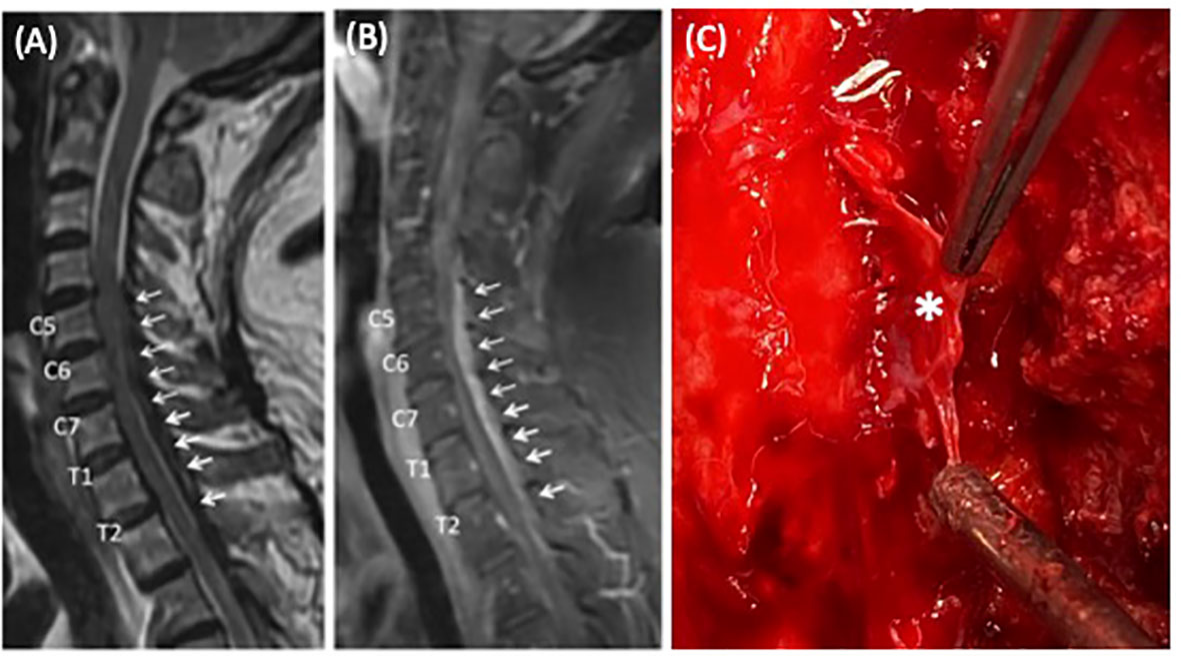
MRI of the cervicothoracic spine demonstrating severe cervicothoracic spinal cord compression and gross intraoperative appearance of the pathology. **(A)** Sagittal short tau inversion (STIR) image. **(B)** Sagittal views of gadolinium-enhanced T1-weighted imaging (T1WI) demonstrating a long-segment, homogenously enhanced tumor lying dorsally, resulting in severe compression to the spinal cord extending from the C5 to the T3 level (*arrows*). **(C)** The grayish sheet-like lesion (held by forceps, *asterisks*) was dissected from the spinal cord.

A 63-year-old female patient with a past medical history pertinent for type 2 diabetes mellitus and hypertension and an unremarkable surgical history presented to the emergency department due to progressive quadriparesis developing over the course of 2 weeks. The patient had no known genetic or autoimmune conditions at presentation. She initially had nuchal and upper back pain that progressed to include right-sided hemiparesis within 10 days, with significant numbness involving both hands. The patient underwent chiropractic and physical therapy; however, her symptoms persisted. On physical motor examination, the strength of the right upper and lower extremity was graded 2/5, while it was 3/5 in the left upper extremity and 4/5 in the left lower extremity. She had a C6 sensory level. Hoffman’s sign was positive bilaterally, as well as Babinski’s sign. Deep tendon reflexes (DTR) revealed hyperreflexia in both lower limbs. CT showed no intracranial hemorrhage or cervicothoracic spine fractures, and an MRI of the cervicothoracic spine (**Figures 1A, B**) demonstrated a dorsal epidural tumor extending from C5 to T3 level, with homogeneous gadolinium enhancement (**Figure 1B**).

**Figure 2:**
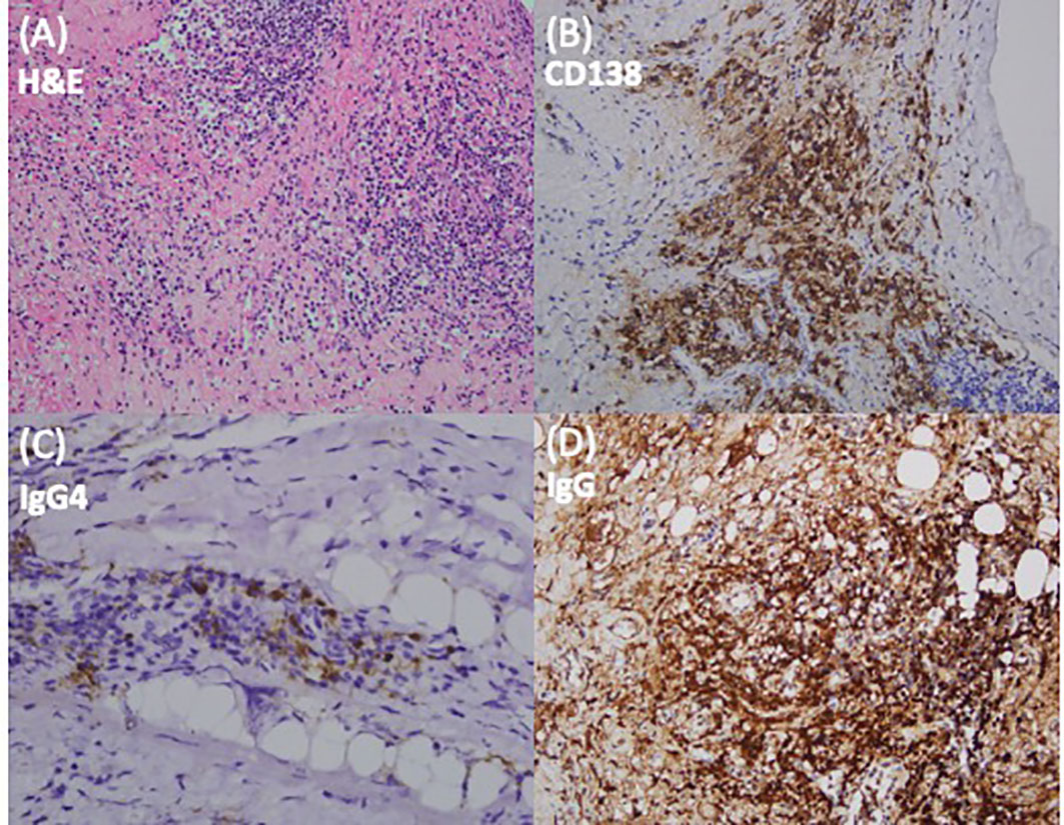
Histopathological findings of the specimens. **(A)** Microscopically, sections of the epidural tissue biopsy show a pseudo-mass formation composed of lymphocytes, plasma cells, focal polymorphonuclear leukocytes, and occasional histiocytes in a background of fibrosis in hematoxylin and eosin staining. **(B–D)** Immunohistochemically, scattered plasma cells are highlighted by CD138, accompanied by a slightly increased ratio of immunoglobulin G4 (IgG4) to IgG+ plasma cells. Based on the histology and immunostaining results, in particular IgG4 immunostaining, IgG4-related disease is suspected. *H&E*, hematoxylin and eosin stain.

The patient was started on systemic steroid therapy (acutely treated with dexamethasone 5 mg, IV Q6H) and subsequently underwent emergent decompressive osteoplastic laminoplasty at C4–C7 and a laminectomy at T1–T3 to achieve gross total removal of the epidural tumor. Grossly, a grayish, soft, and sheet-like tumor (**Figure 1C**) was identified upon laminectomy. Intraoperative frozen sections showed nodular aggregation of lymphoplasmacytic cells. The pathology report revealed that the pseudo-mass formation was mainly composed of lymphocytes, plasma cells, focal polymorphonuclear leukocytes, and occasional histiocytes in the background of fibrosis (**Figure 2A**). Immunohistochemically, there were scattered plasma cells highlighted by CD138, accompanied by a slightly increased ratio of immunoglobulin G4 to immunoglobulin G (IgG4/IgG) (**Figures 2B–D**). Based on the histology and immunostaining findings, in particular the IgG4 staining, IgG4-related hypertrophic pachymeningitis (IgG4-RHP) was suspected.

**Figure 3:**
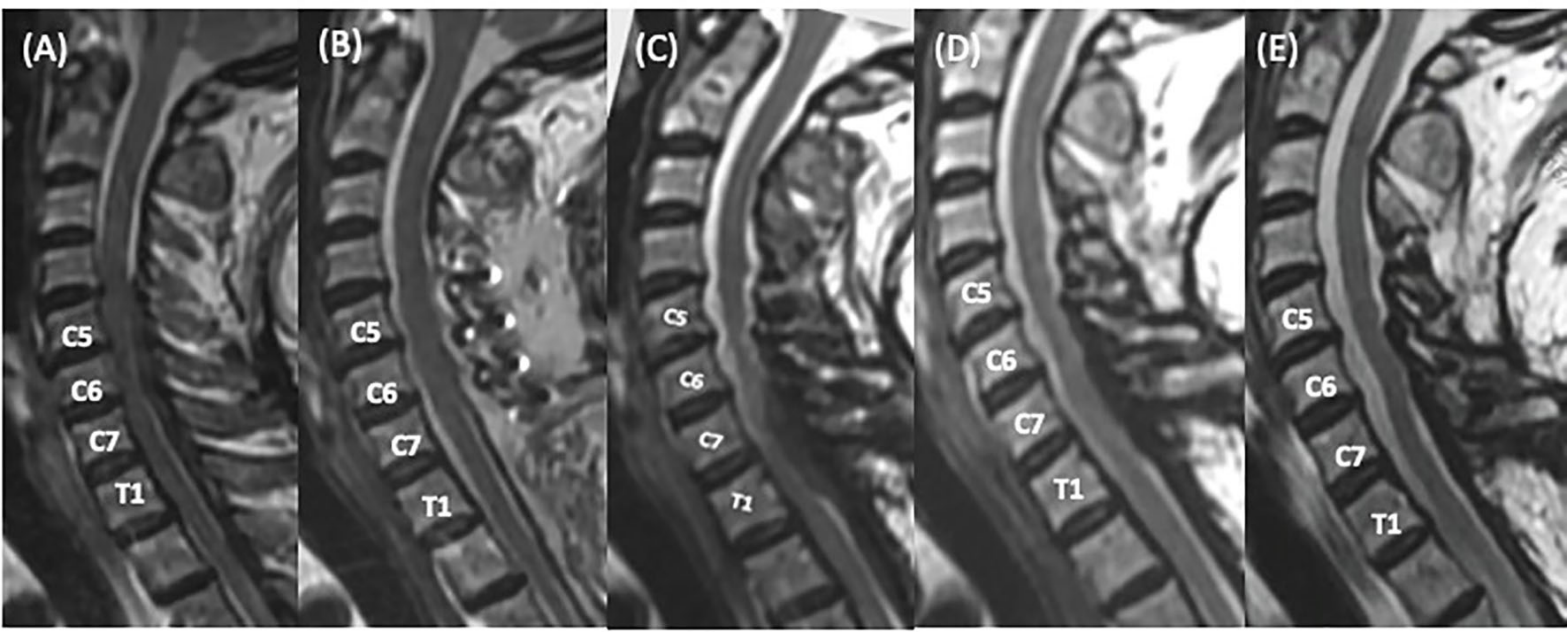
Serial Sagittal short tau inversion sequences (STIR) of the cervicothoracic spine obtained demonstrating disease progression and response to therapy. **(A)** Pre-operation. There is a severe spinal cord compression from a homogenoulsy hypointense epidural tumor, traversing the C5 to the T3 level. **(B)** At 2 weeks post-operation. The tumor was completely removed. **(C)** At 9 months post-operation. After 5 months of steroid discontinuation, there is a recurrence of immunoglobulin G4-related hypertrophic pachymeningitis (IgG4-RHP), resulting in spinal cord compression, especially at the C6 to the T1 level. Systemic steroid was reinitiated. **(D)** At 12 months post-operation. Azathioprine was added 3 months after systemic steroid was reinitiated. **(E)** The recurred lesion continued to regress and stabilized under medical treatment.

**Figure 4:**
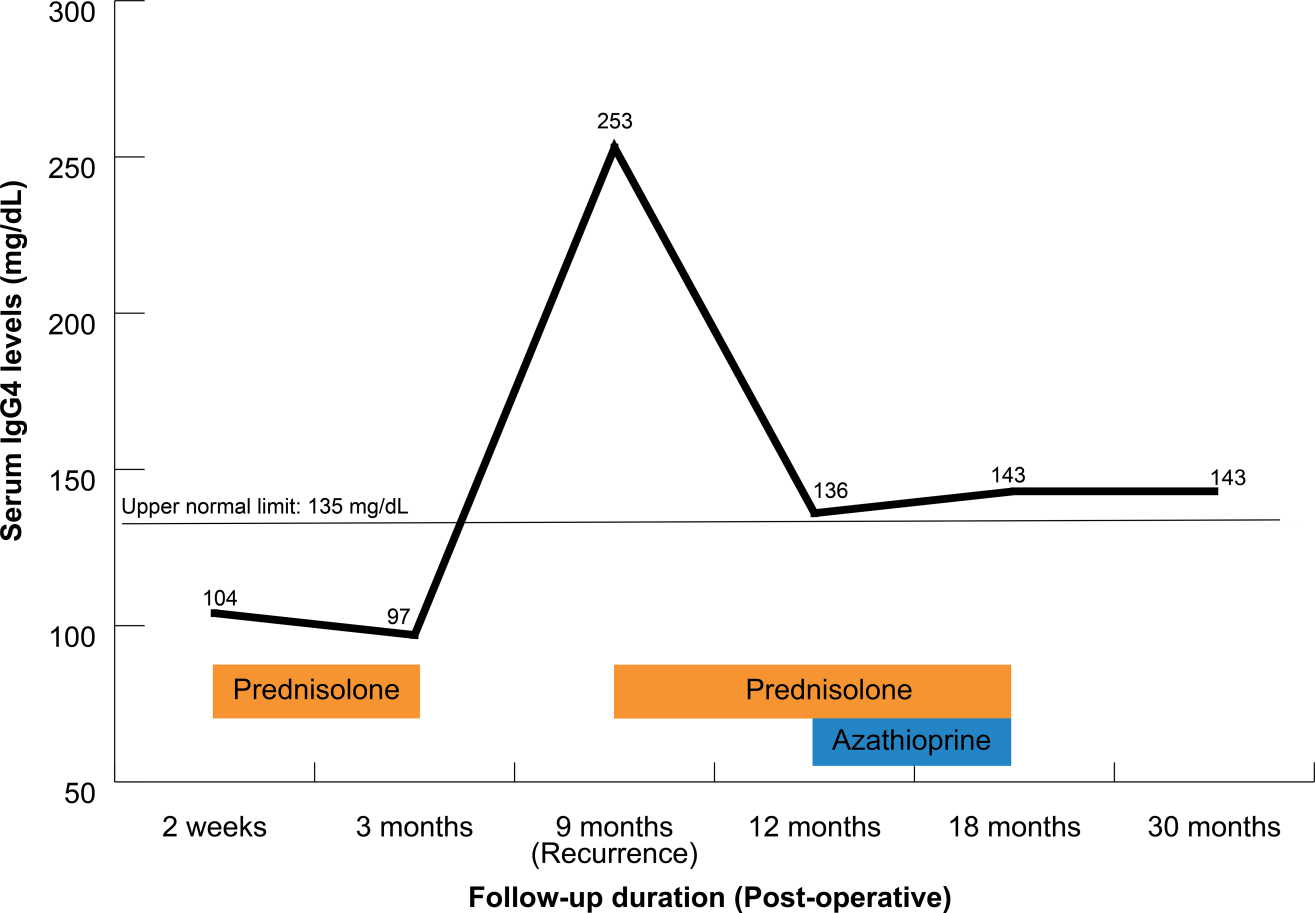
Trend of serum immunoglobulin G4 (IgG4) concentrations throughout the treatment course. Preoperative IgG4 levels were not assessed as IgG4-related disease (IgG4-RD) was not initially suspected. The serum IgG4 level did not exceed 135 mg/dl until the first documented disease recurrence. *Horizontal line* indicates the upper normal limit of serum IgG4 level (135 mg/dl). *Horizontal bars* represent the duration of corticosteroid and azathioprine therapy. *Pred*, prednisolone; *Aza*, azathioprine.

The patient endorsed a near-complete recovery in motor function after the surgery, as well as a marked improvement in her symptoms of pain and numbness. Compared with the preoperative MRI (**Figure 3A**), the follow-up MRI at 2 weeks postoperatively confirmed gross total resection of the tumor (**Figure 3B**). Under the impression of IgG4-RHP, maintenance therapy with prednisolone (5 mg, BID PO) was initiated. However, the treatment had to be paused at the 4-month mark after initial presentation due to the development of cushingoid features, such as a dorsocervical fat pad (buffalo hump), moon facies, limb edema, purple abdominal striae, bloating, and poor glycemic control. At 5 months after stopping maintenance therapy with systemic steroids (prednisolone 5 mg, BID PO), and roughly 9 months out from her initial presentation, the patient presented with a recurrence of paresthesias in both hands and a spastic gait. A serial follow-up of the serum IgG4 concentration revealed a significant increase (**Figure 4**) after the discontinuation of prednisolone.

A series of examinations were conducted to rule out the involvement of other organs typically affected by IgG4-related disease (IgG4-RD), including the lacrimal glands, the salivary glands, the pancreas, and other intra-abdominal organs. The list of differential diagnoses included other autoimmune diseases such as Sjogren’s syndrome, primary sclerosing cholangitis, anti-neutrophil cytoplasmic antibody (ANCA)-associated vasculitis, and large vessel vasculitis, which were all screened for and ruled out. **Table 1** provides a list of the various differential diagnoses and the key clinical and diagnostic clues. A whole-body positron emission tomography–computed tomography (PET-CT) scan at this time showed an increased 18F-fluorodeoxyglucose (18F-FDG) uptake in the cervicothoracic spine, which is suggestive of recurrent IgG4-RHP. MRI scans of the cervicothoracic spine without contrast (**Figure 3C**) confirmed tumor recurrence. Systemic steroid therapy (prednisolone 5 mg, PO BID) was reinitiated. At an outpatient clinic follow-up, azathioprine (50 mg, PO daily) was added to the maintenance regimen as a cost-effective oral steroid-sparing immunomodulatory agent. The dosage was increased to 75 mg daily a month later, while the steroid dosage was gradually tapered off. The patient tolerated the regimen and did not endorse any gastrointestinal side effects, a common limiting factor of treatment with azathioprine. Of note is that rituximab in general is the preferred steroid-sparing agent due to its significantly lower frequency of refractory disease compared with other conventional immunomodulatory agents (31%) (8, 9). The patient reported improvement in spasticity and the numbness in her hands at a 3-month follow-up visit in the outpatient clinic. The serum IgG4 concentration levels also normalized, and a follow-up MRI scan (**Figures 3D, E**) showed regression of the lesion.

**Table 1:** Key differential diagnoses of IgG4-related pachymeningitis.

Etiologic category	Clinical/laboratory clues	Diagnostic clues and implications
Idiopathic hypertrophic pachymeningitis	Chronic headache and cranial neuropathies without systemic disease	Diagnosis of exclusion after infection, neoplasm, and autoimmune causes are investigated; often responsive to steroids 1, 2
IgG4-related pachymeningitis	Prior IgG4-RD or other organ involvement; increased IgG4 plasma cells on biopsy	Biopsy shows storiform fibrosis and IgG4+ plasma cells; often benefits from steroids and B-cell therapies in refractory cases 1, 3
Granulomatous disease (Neurosarcoidosis, GPA)	Systemic sarcoid manifestations, pulmonary involvement, ANCA positivity for GPA	Imaging nodular pachymeningeal enhancement and supportive systemic pathology guide immunosuppression selection 2, 4
Infectious causes (tuberculosis, syphilis, other)	Fever, CSF culture/PCR positivity, systemic infectious signs	Microbiologic confirmation directs antimicrobial therapy rather than primary immunosuppression 5, 6
Neoplastic infiltration or dural metastasis	Known malignancy or imaging features suggesting mass or invasion	Biopsy or comprehensive oncologic workup required; treatment directed at malignancy and possible neurosurgical decompression 7

ANCA, Anti-neutrophillic antibodies; CSF,Cerebrospinal fluid; GPA, Granulomatosis with Polyangitis; PCR, polymerase chain reaction.

There remains a paucity of studies looking into the natural course of IgG4-RHP and its treatment. This is in part due to cases of IgG4-RD being extremely rare, more so those with meningeal involvement. Our case illustrates a complete clinical course, with clear imaging documentation corroborating the effects of surgical decompression of the spinal cord and medical treatment with steroid therapy in combination with a steroid-sparing immunomodulatory agent for IgG4-RHP, especially in cases of disease recurrence. Prior studies that have looked into pachymeningitis have primarily focused on medical management (9, 10).

A 2022 case series, however, detailed the progression of disease in two adults and two adolescents who were found to have IgG4-RHP requiring some form of surgical intervention, albeit in the cranium (11). The clinical symptoms were epilepsy (*n* = 2), ataxia and nausea (*n* = 1), and facial nerve palsy (*n* = 1). MRI studies showed contrast enhancing lesions in the temporal region in two patients and in the cerebellar region in the other two patients. Subtotal resection was performed in two instances, and a biopsy via a suboccipital retrosigmoid approach was obtained in the other two patients. Both histochemical and immunohistochemical analyses revealed IgG4-RD in all four patients. Immunomodulatory therapy with a regimen that consisted of a steroid (in all four cases) with some combination of either methotrexate, rituximab, or both led to clinical stability over a 5-year follow-up interval in all four cases (11).

## Discussion

IgG4-RD is an immune-mediated fibroinflammatory condition that can affect multiple organs (12). It is typified by several characteristic features, which include tumor-like swelling, a lymphoplasmacytic infiltrate enriched in IgG4-positive plasma cells, and a variable degree of fibrosis that typically carries a characteristic “storiform” pattern (13–15). When the meninges are involved, and more specifically the pachymeninges, it is known as IgG4-related hypertrophic pachymeningitis (IgG4-RHP). The hypertrophic dura mater by mechanical compromise leads to the subsequent symptoms, which vary from headaches and cranial nerve palsies to motor or sensory deficits (16), depending on the location of the lesion and the nerves compressed. It can be difficult to distinguish IgG4-RHP-related lesions from other types of space-occupying lesions in the central nervous system simply by the presenting symptoms.

In general, non-contrast CT scans often offer limited clues, unless the adjacent bone is involved. A thickened hypertrophic dura may be visible after the administration of iodine contrast in CT scans. On MRI studies, IgG4-RHP may present as linear dura thickening or a bulging mass (16), which is typically isointense to bone in T1-weighted imaging and hypointense in T2-weighted imaging. Gd contrast-enhanced MRI may reveal a homogenous but ill-defined enhancement of the lesions (17), which need to be differentiated from infectious processes such as an abscess or tuberculosis, an inflammatory fibroblastic tumor, lymphoma, granulomatous with polyangiitis, giant cell arteritis, Langerhans’s cell histiocytosis, sarcoidosis, or malignancies (18). **Table 1** provides some of the key differential diagnoses and clues to distinguish between them.

In patients with IgG4-RD or with IgG4-RHP, elevated serum IgG4 or IgG concentrations are observed in 55%–97% of patients (16, 19). Therefore, the use of only serum IgG4 titers lacks the necessary sensitivity and specificity to provide a reliable diagnosis (16, 20, 21). When IgG4-RHP is present, an elevated serum IgG4 level typically suggests involvement of other organs apart from the meninges alone, while the serum IgG4 concentration may remain normal in spite of elevated local IgG4 concentration levels at the lesion site, particularly when the meninges are the only site of the disease (16).

The diagnosis of IgG4-RD should be based on specific histopathological findings, typical laboratory and radiological aspects, and an appropriate clinical context (22). In 2011, two IgG4-RD study groups were organized by the Japanese government to establish comprehensive clinical diagnostic criteria for IgG4-RD (21). These include: 1) clinical examination showing characteristic diffuse/localized swelling or masses in single or multiple organs; 2) serum IgG4 concentration >135 mg/dl; and 3) histopathological findings characteristic of IgG4-RD (as mentioned above). In our case, IgG4-RHP was not suspected until the pathology examination results offered such clues; therefore, the checkserum IgG4 concentration was not assayed until 1 week after systemic steroid therapy had been initiated, which may have resulted in a suppression of the initial serum IgG4 concentration (104 mg/dl) recorded. After pausing steroid therapy at the 4-month mark, the serum IgG4 concentration peaked at 253 mg/dl (when measured again at the 9-month interval), at which time the patient was diagnosed with disease recurrence (**Figure 4**).

In 2012, Deshpande et al. (14) issued a consensus statement on the pathological examination of IgG4-RD, which endorses a three-tier terminology system for its histopathological diagnosis: highly suggestive, probable, or insufficient. Surgical specimens with two out of three histopathological features (i.e., dense lymphoplasmacytic infiltrates, fibrosis, or obliterative phlebitis) and >40% of IgG4-to-IgG+ plasma cell ratio are “highly suggestive” of IgG4-RD. Cases with “probable” histological features of IgG4-RD typically fall into the following sub-categories: 1) specimens with only one out of three histological features; 2) specimens that are acquired by needle biopsy and with insufficient histological evidence; and 3) specimens of meninges, skin, or other organs with limited published data of IgG4-RD involvement. Our case fits into the first and third sub-categories of a surgical specimen with probable features of IgG4-RD. Only one of the histopathological features needed in diagnosing IgG4-RD was present, and there is a paucity of published articles on IgG4-RHP, which made it more difficult to definitively diagnose IgG4-RHP.

Excluding malignancies such as solid organ cancer or lymphoma is necessary when IgG4-RD is suspected clinically (12). Other autoimmune diseases, such as Sjogren’s syndrome, primary sclerosing cholangitis, Castleman’s disease, secondary retroperitoneal fibrosis, granulomatosis with polyangiitis (previously known as Wegener’s granulomatosis), sarcoidosis, and eosinophilic granulomatosis with polyangiitis (previously known as Churg–Strauss syndrome), also pose confusion in correctly identifying IgG4-RD (21). As such, a tissue biopsy for histopathological examination is always strongly recommended (23).

Steroids comprise the first-line treatment for IgG4-RD (24), with an initial dosage of 30–40 mg/day or 0.67 mg/kg^−g^/day^−a^ for at least 2–4 weeks, which may be tapered off gradually afterward. The optimal duration of maintenance therapy has not been evaluated rigorously and appears to be dependent on several patient-specific factors (23). Steroid monotherapy is generally accepted as the first-line treatment. There are no clinical trials or data supporting the efficacy of steroid-sparing immunomodulatory agents such as rituximab, azathioprine, mycophenolate mofetil, tacrolimus, or methotrexate as the choice of monotherapy (16, 23), let alone as an add-on therapy (23). The MITIGATE trial, however, is a landmark phase 3 clinical study that led to the approval of inebilizumab as the first-ever Food and Drug Administration (FDA)-approved treatment for IgG4-RD. It was found to reduce the risk of flares and increase the likelihood of flare-free complete remission at 1 year (25). The medication is still not readily available. Low-dose prednisolone as maintenance therapy may lower the risk of disease recurrence (24), but also leads to a higher risk of treatment-related morbidity (26). The introduction of a steroid-sparing immunomodulatory agent for continuation in the remission maintenance period has been suggested in disease recurrences (23).

For patients presenting with neurological deficits and a mass-like lesion in the nervous system, with no history or clinical clues indicating IgG4-RD, it can be difficult to take IgG4-RHP into consideration and initiate systemic steroid therapy from the onset. An additional challenge is making the decision of medical management with systemic steroid therapy alone instead of performing a decompression surgery, especially when the meninges are the sole site of involvement. In our case, emergent surgical decompression of the spinal cord was unavoidable in an effort to preserve neurological function.

After treatment, monitoring of the serum IgG4 concentration may be useful in tracking disease activity, but should not be used as the sole predictor (27). Of note is that the serum IgG4 level does not return to normal levels even after disease remission in 63% of patients and does not rise above the normal range after disease recurrence in 10% of patients (27). Aside from the serum IgG4 concentration, PET-CT with 18F-FDG has been proven a useful and sensitive modality to detect disease activity (28).

## Conclusion

This case illustrates the complete clinical course of IgG4-RHP, including surgical decompression and subsequent medical management with steroid therapy along with an immunomodulatory agent, and is supported by comprehensive imaging documentation of the treatment responses and disease recurrence. Although meningeal involvement in IgG4-RD is uncommon, it should be considered in the differential diagnosis of a long-segment, homogeneously enhancing epidural mass. Surgical decompression is indicated in the setting of acute neurological deterioration. Serum IgG4 titers and 18F-FDG PET-CT may be useful in assessing the disease activity and therapeutic response. Systemic steroid therapy remains the first-line therapy for IgG4-RHP, while immunomodulatory agents may be required in cases of steroid intolerance or recurrence and as an adjuvant.

## Data Availability

The original contributions presented in the study are included in the article/supplementary material. Further inquiries can be directed to the corresponding author.
